# Effects of Host-rock Fracturing on Elastic-deformation Source Models of Volcano Deflation

**DOI:** 10.1038/s41598-017-10009-6

**Published:** 2017-09-08

**Authors:** Eoghan P. Holohan, Henriette Sudhaus, Thomas R. Walter, Martin P. J. Schöpfer, John J. Walsh

**Affiliations:** 1 UCD School of Earth Sciences, University College Dublin, Dublin, Ireland; 2 GFZ-German Research Centre for Geosciences, Section 2.1 - Physics of Earthquakes and Volcanoes, Potsdam, Germany; 3 Institute of Geosciences, University of Kiel, Kiel, Germany; 4 Department for Geodynamics and Sedimentology, University of Vienna, Vienna, Austria

## Abstract

Volcanoes commonly inflate or deflate during episodes of unrest or eruption. Continuum mechanics models that assume linear elastic deformation of the Earth’s crust are routinely used to invert the observed ground motions. The source(s) of deformation in such models are generally interpreted in terms of magma bodies or pathways, and thus form a basis for hazard assessment and mitigation. Using discontinuum mechanics models, we show how host-rock fracturing (i.e. non-elastic deformation) during drainage of a magma body can progressively change the shape and depth of an elastic-deformation source. We argue that this effect explains the marked spatio-temporal changes in source model attributes inferred for the March-April 2007 eruption of Piton de la Fournaise volcano, La Reunion. We find that pronounced deflation-related host-rock fracturing can: (1) yield inclined source model geometries for a horizontal magma body; (2) cause significant upward migration of an elastic-deformation source, leading to underestimation of the true magma body depth and potentially to a misinterpretation of ascending magma; and (3) at least partly explain underestimation by elastic–deformation sources of changes in sub-surface magma volume.

## Introduction

The underlying cause(s) of deformation at volcanoes may be investigated by comparing surface displacement patterns to solutions obtained from analytical or numerical models that contain one or more deformation sources^1–3^. The attributes of such sources may include shape, size, and orientation, as well as changes in volume or pressure. These attributes can in turn be interpreted in terms of the properties of subterranean magma bodies or pathways. A number of analytical^4–7^ and numerical^8–12^ source modelling approaches are now customarily used to help understand the past, current and future behaviour of active volcanoes^2, 13–16^. As computing power advances, such modelling is increasingly used in real-time hazard assessment at volcanoes^16^.

Interpreting such source models can be problematic with progressive deformation that is complex in space and time, however. This is partly because the surface displacements produced by such a model are a function of not only the deformation source attributes, but also the assumptions made about the medium hosting the source. An assumption of many source models is that the host-rock deformation is spatially continuous and linearly elastic. This assumption is reasonable as long as the related strains are ‘small’ – i.e. ~1–2%^17^. Field, geodetic and seismic evidence indicates, however, that episodes of volcano unrest or eruption may entail substantial non-elastic deformation^4, 18^. Recent modelling approaches based on continuum mechanics have consequently included elasto-plastic^19^,^20^ or viscoelastic^21, 22^ host-rock rheologies.

Continuum-based approaches nonetheless face limitations when the strains characterising volcano deformation are large and discontinuous, such as occur with substantial host-rock fracturing^4, 23, 24^. While displacements and strains during a volcano inflation episode are generally small, they can become very large during a volcano deflation episode. Deflation can in this case progress to a collapse of the host-rocks^25, 26^, which can occur exclusively underground^27^ or with formation also of a caldera at the surface^28, 29^. This progression from low-strain inflation to high-strain collapse is exemplified by activity at Piton de la Fournaise volcano in 2007. Continuum-based elastic modelling of surface displacements during that activity revealed intriguing changes in source attributes with time^30^, as summarised below.

We tested the hypothesis that such changes in the elastic-deformation source inferred for the March-April 2007 activity at Piton de la Fournaise are related to the formation and propagation of collapse-related fracture systems^30^. Although elastic dislocation models have successfully explained complex, fracture-related surface displacement patterns at other volcanoes^31, 32^, fracture location and geometry is largely predefined in such studies. The basis of our test is the use of a two-dimensional Distinct Element Method (DEM) model that explicitly simulates the emergent growth and development of brittle fracture systems through both elastic and inelastic (frictional-plastic) deformation^33^. To our knowledge, this paper presents the first reported use of the DEM for interpreting surface displacements in nature and their elastically-modelled sources.

## Piton de la Fournaise volcano: 2007 eruptive activity and related elastic-deformation sources

**Figure 1:**
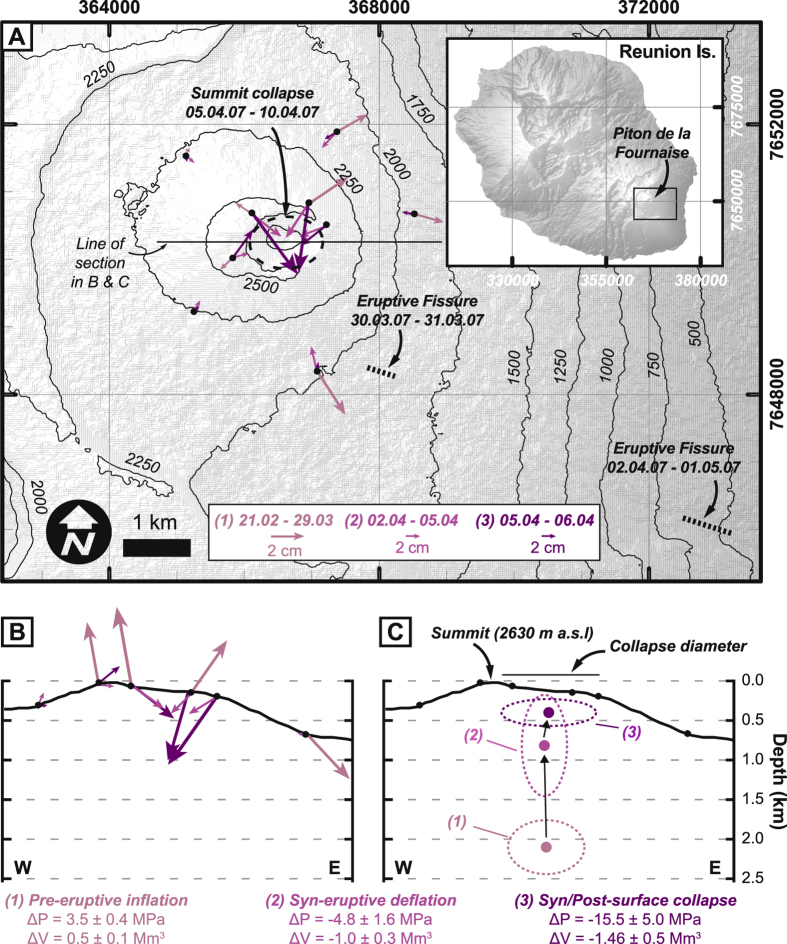
Volcano inflation-deflation with concurrent changes in the location and geometry of a simple elastic-deformation source. (**A**) Map of GPS-measured surface displacements associated with pre-eruptive inflation, syn-eruptive deflation and surface collapse during the March-April 2007 activity at Piton de la Fournaise volcano, Reunion Island. Hill-shaded relief of Reunion Island is derived from an ASTER GDEM v2 digital elevation model, a product of NASA and METI (https://lpdaac.usgs.gov/dataset_discovery/aster). Black lines are topographic contours in metres above sea level derived from the ASTER GDEM. Grid co-ordinates are for Universal Transverse Mercator projection (RGR 1992 UTM 40 S). Map made with ArcMAP 10.2.2 (http://desktop.arcgis.com/en/arcmap/) and refined in Adobe Illustrator CS6 (http://www.adobe.com/products/illustrator.html). Displacement data are from^30^. Note the change in scale of displacement vectors from the inflation phase to the deflation phases. (**B**) Surface displacements projected into an E-W section view. Arrow scales are same as that in part A. (**C**) Ellipsoidal elastic - deformation sources constrained for each of these three phases by past continuum-based (Boundary Element Method) modelling of the surface displacements^30^. Positive changes in source pressure (ΔP) and volume (ΔV) before the eruption become increasingly negative during the eruption. Overall, the position of the optimized source moved toward the surface with time. Also the shape of the source, which was fixed to be either horizontal or vertical, changed from sill-like to stock-like and vice versa.

Piton de la Fournaise is one of the world’s most active volcanoes, and its March-April 2007 basaltic eruption is a well-constrained example of inflation and deflation during volcanic unrest^30, 34–37^ (Fig. 1A,B). With a dense rock equivalent (DRE) lava volume of 150 × 10^6^ m^3^, the 2007 eruption was one of the largest ever recorded at this volcano^38^. It represented the culmination of a longer-term inflationary period beginning in 1998 and punctuated by several smaller eruptions^39, 40^. The 2007 eruption also occurred with a complex seaward motion of the volcano flank^41, 42^, but the flank displacements are largely distinct from the summit area displacements, which are the focus of this study.

From June 2003–April 2007, the summit of Piton de la Fournaise cumulatively uplifted by about 1 m^30, 40, 43^. Immediately prior to eruption, between February 21^st^ to March 29^th^ 2007, displacement of up to 0.03 m occurred (Fig. 1A,B). Modelling of these displacements with the Boundary Elements Method, which assumed linear elasticity, yielded a slightly horizontally elongated deformation source at ~2 km below the volcano summit^30^ (Fig. 1C).

Upon the lateral intrusion of magma and the onset of eruption on March 31^st^ 2007, the volcano began to deflate. Summit displacements of up to 0.07 m occurred between April 2^th^–5^th^ (Fig. 1A,B). The elastic-deformation source geometry associated with this initial syn-eruptive deflation changed shape to vertically-elongated (‘stock-like’) and moved markedly upward to ~1 km below the volcano summit (Fig. 1C)^30^. Also during this phase, the number of volcano-tectonic earthquakes, which are typically associated with host-rock fracturing^44^, increased progressively below the edifice^34^.

A subsequent phase of more rapid deflation associated with a summit caldera collapse occurred on April 5^th^–6^th^. Displacements of up to 0.07 m were measured immediately outside the caldera (Fig. 1A,B), but the caldera floor subsided by about 340 m. The displacements outside the caldera were linked with an elastic-deformation source that was markedly horizontally elongated (‘sill-like’) and located at only ~300 m below the volcano summit (Fig. 1C)^30^. Since the 2007 eruption, and until 2014, the summit area has cumulatively deflated by 0.5–1 m^45, 46^.

## Modelling Approach

Our approach comprises two steps: (1) forward modelling of volcano deflation with the DEM and (2) inverse modelling of the DEM-displacements with an analytical solution for a deformation source in a linear elastic half-space. The set-up for both approaches is shown in Supplementary Figure S1 (see Methods for details). We focus first on deformation and displacements in a DEM model with a pre-deflation geometry similar to what is thought to have existed prior to the March-April 2007 eruption at Piton de la Founaise, as constrained by geodetic, seismological and petrological evidence^30, 38, 39^. We run this gravitationally-loaded model under conditions where host-rock fracturing is either permitted or prohibited. By means of the analytical modelling, we then ‘blindly’ invert (or optimize) for the apparent deformation source properties that best reproduce the DEM-derived surface displacements.

The elastic-deformation source here comprises two mutually-perpendicular rectangular dislocation planes that are set normal to the plane of the DEM model (see Methods). This source model can be considered equivalent to a triaxial cavity of infinitesimal^4, 22^ or finite^47^ size. To help visualize how the two-dislocation source might relate to the shape of such an enclosed sub-surface body (or a ‘volumetric deformation source’), we define a ‘strength ellipse’ (see Methods). It is important to emphasize here that the objective of the second modelling step is not necessarily to retrieve precisely the properties of the DEM magma body. The objective is rather to see how an elastic-deformation source is affected by the combination of mechanical processes (magma body depletion, host-rock deformation) simulated in the DEM model. Thus our study aims to provide a basis for an improved geological interpretation of routinely-performed source modelling at volcanoes.

## Modelling Results

**Figure 2:**
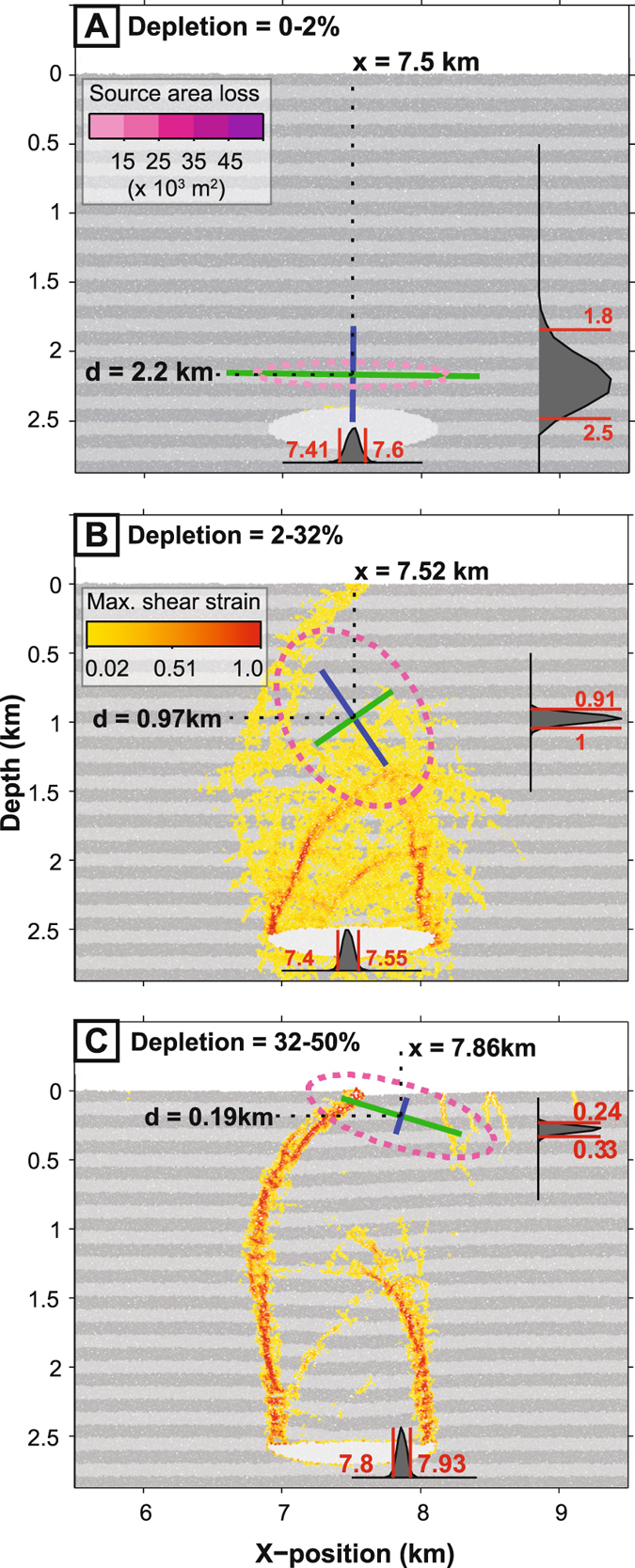
Effects of deflation-related host-rock deformation on an elastic-deformation source for incremental displacements. (**A**) Low-strain deformation. Superimposed upon the DEM model is the optimal elastic-deformation source, for which the dark-grey histograms represent likelihood distributions of its depth and lateral position. The strength ellipse colour indicates the area loss due to closing across the source’s planes (i.e. the source’s ‘potency’). (**B**) Subsurface host-rock fracturing and onset of surface collapse. (**C**) Surface collapse and focussing of deflation-related strain onto through-going fractures. Note that progressive development of host-rock fracturing leads to upward migration and tilting of the elastic-deformation source.

Figure 2 shows a close up of the DEM model at three stages of deflation: (a) an initial low-strain stage after small magma body depletions (0–2%), (b) an intermediate stage in which host-rock fracturing develops mainly underground above the magma body, but has just reached the surface (2–32% depletion); and (c) an advanced stage in which host-rock fracturing has broken through to surface and developed major faults - a ring fault system in three-dimensions - that delimit a collapse caldera (32–50% depletion). Superimposed on the DEM model is the optimal elastic-deformation source and its strength ellipse for that increment of depletion.

For the initial low-strain stage (Fig. 2A **)**, the likelihood distribution of the elastically-modelled source solutions overlaps with the true depth and lateral position of the DEM model reservoir at 95% confidence. The horizontal plane in the source has a much greater area change than the vertical plane – this is reflected in a strength ellipse that, like the DEM magma body, is horizontal and ‘sill-like’ in shape. For the intermediate-strain stage (Fig. 2B), upward propagation of subsurface fracturing causes the elastic-deformation source to decrease in depth and to become markedly inclined. Also, the strength ellipse becomes less sill-like. As the host-rock deformation approaches and breaks through to surface, the strength ellipse becomes more vertically than horizontally elongated – i.e. it becomes ‘stock-like’ in shape. For the advanced high-strain stage (i.e. with surface collapse) (Fig. 2C), the elastic-deformation source rises still further to lie just below the surface. The greater area change occurs now on the more gently-inclined of the two planes; the strength ellipse reverts to being horizontally-elongated and ‘sill-like’ in shape.

**Figure 3:**
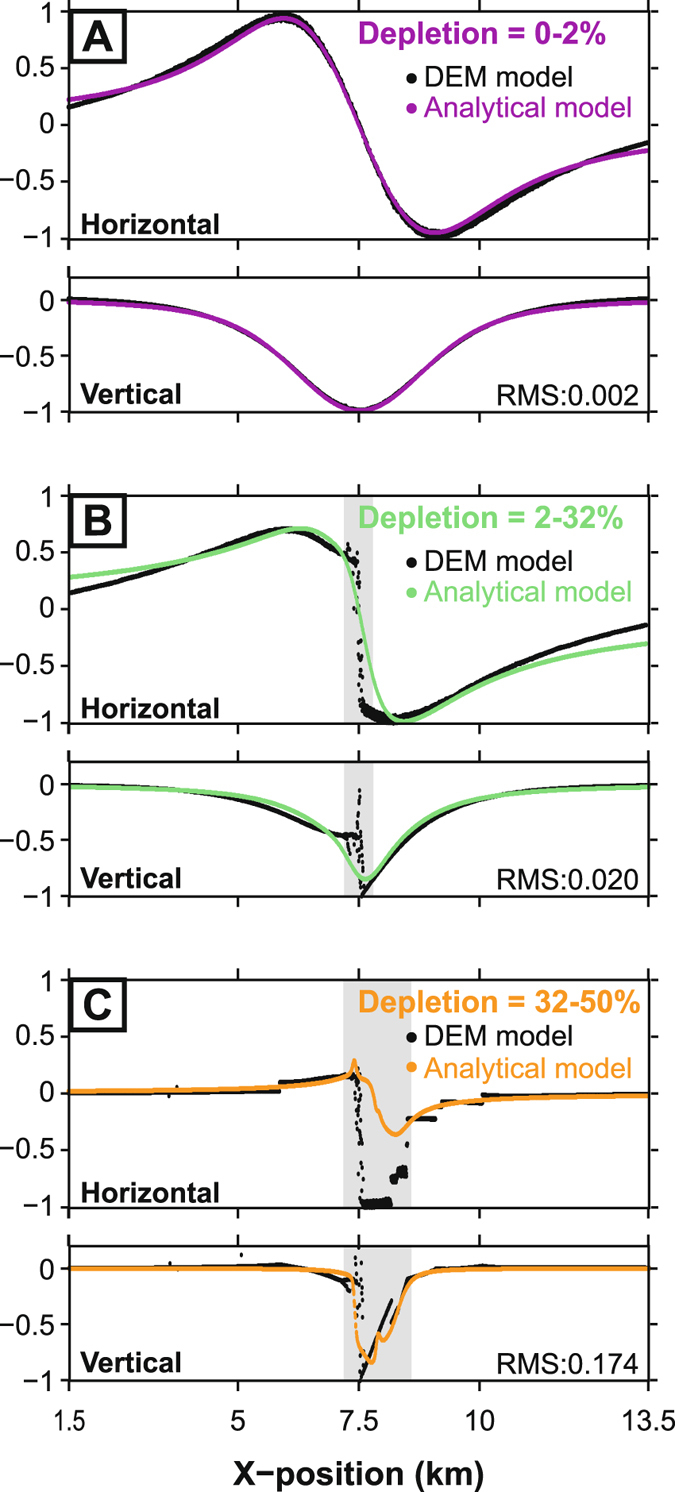
Profiles of incremental surface displacement from the DEM model and the optimum elastic - deformation source model. The horizontal (top) and vertical (bottom) components are plotted for (**A**) low-strain deflation (0–2% depletion), (**B**) high-strain subsurface deformation and onset of surface collapse (2–32% depletion) and (**C**) surface collapse (32–50% depletion). Profiles are nomalised to the absolute maximum or minimum of that component. The grey shaded areas denote parts of the DEM surface displacement profiles that were excluded from elastic source modelling (see Methods for details). The misfit of the optimized source displacements to those of the DEM models is indicated by the normalized root mean squared (RMS) error.

Figure 3 shows the incremental surface displacement profiles of the DEM model and the optimum elastic-deformation source at each of the above stages. With a root mean square (RMS) error of 0.002, the fit of the elastic-deformation source displacements to those of the DEM is very close in the initial low-strain stage (Fig. 3A). As host-rock fracturing develops, the RMS error progressively increases (Fig. 3B,C); this reflects the increasing difficulty of capturing complex deformation with a simple elastic-deformation source.

**Figure 4:**
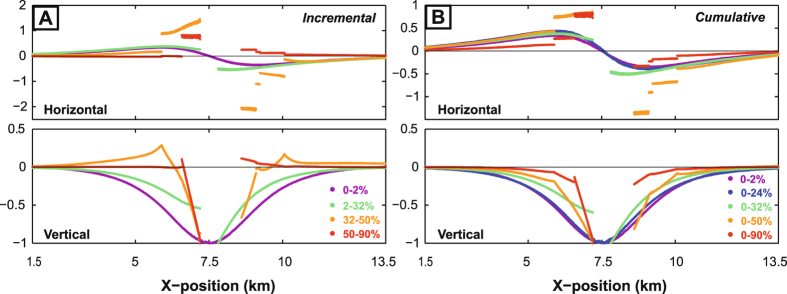
Evolution of surface displacement profiles in the DEM model. (**A**) Incremental displacements; (**B**) Cumulative displacements. All values are normalized by the maximum vertical displacement. Gaps in the centre of some profiles arise from exclusion of data from the collapse zone of pronounced surface fracturing. Minor peripheral fractures are as seen as steps or kinks in some of the profiles. Note: (1) the progressive sharpening of both horizontal and vertical displacement profiles with depletion; and (2) the general increase in the ratio of horizontal to vertical displacement (visible in the upper plots) from 0–50% depletion followed by a slight decrease from 50–90% depletion.

Figure 4 enables closer comparison of the evolution of both incremental and cumulative DEM displacement profiles. Firstly, the horizontal and vertical profiles both become sharper – i.e. gradients increase closer to the center - as depletion and host-rock strain increase, especially once fracturing breaks through to the surface. The sharpening of the profiles after surface collapse is in part due to a subtle rebound of the peripheries of the collapsing area (note positive displacement in Fig. 4A). Secondly, the ratio of horizontal to vertical displacement increases as fracturing migrates upward, peaking at in the early stage of collapse and then decreasing. For incremental displacement, the ratio of maximum horizontal to maximum vertical displacement, H_max_/V_max_ = 0.38, 2.0 and 0.9 at depletions of 2%, 50% and 90%, respectively. As discussed below, these changes in profile sharpness and horizontal/vertical displacement ratio lead to the shallowing and shape change of the elastic-deformation source. Thirdly, asymmetric strain accumulation in the DEM leads to notably asymmetric surface displacement profiles and hence tilted elastic-deformation sources.

**Figure 5:**
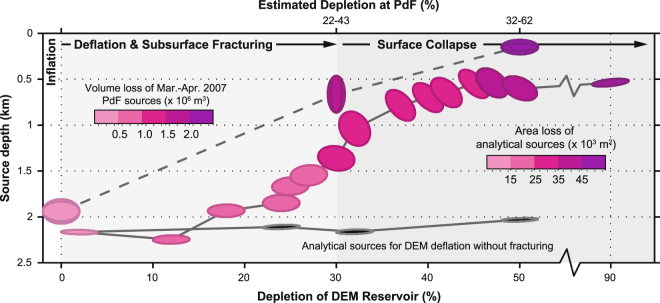
Evolution of elastic-deformation source attributes for cumulative displacements. Plotted are the strength ellipses and depths of optimum deformation sources for fractured (pink-lilac) and non-fractured (black) DEM models against increased DEM magma reservoir deflation (bottom axis). Shown for comparison are the depth and shape of the sources resolved for the inflation and deflation stages of the March-April 2007 activity at Piton de la Fournaise plotted against the estimated percentage of magma extracted (i.e., intruded and erupted) at each stage (top axis). Note that the depletion estimate at Piton de la Fournaise is subject to considerable uncertainty, depending on the approach used for estimation^30, 35^, and may be much less than indicated here^37^. Note also that the sources for Piton de la Fournaise have double-framed ellipses in order to show both cross-sectional profiles through what are triaxial ellipsoids^30^.

Figure 5 shows how independent optimization results for cumulative displacements over a wider range and a greater number of depletion stages. The changes in depth and shape of the elastic-deformation source are slightly less pronounced in the cumulative cases than in the incremental cases shown in Figs 2 and 3, and the RMS error is lower, especially for the advanced surface collapse stage (50% depletion) (see Supplementary Figures S7 and S8). Nonetheless, the source depth again progressively decreases, and the strength ellipse progressively changes from sill-like to stock-like and back to sill-like. Moreover, the tilt of the source is gradually reduced or even reversed in the latest stages of collapse (50–90% depletion), as new faults lessen the asymmetry in host rock deformation (see Supplementary Figure S7). Figure 5 also shows that when host-rock fracturing is not permitted, the depth, orientation and strength ellipse of the elastic-deformation source remain essentially unchanged as depletion increases (see also Supplementary Figure S4).

**Figure 6:**
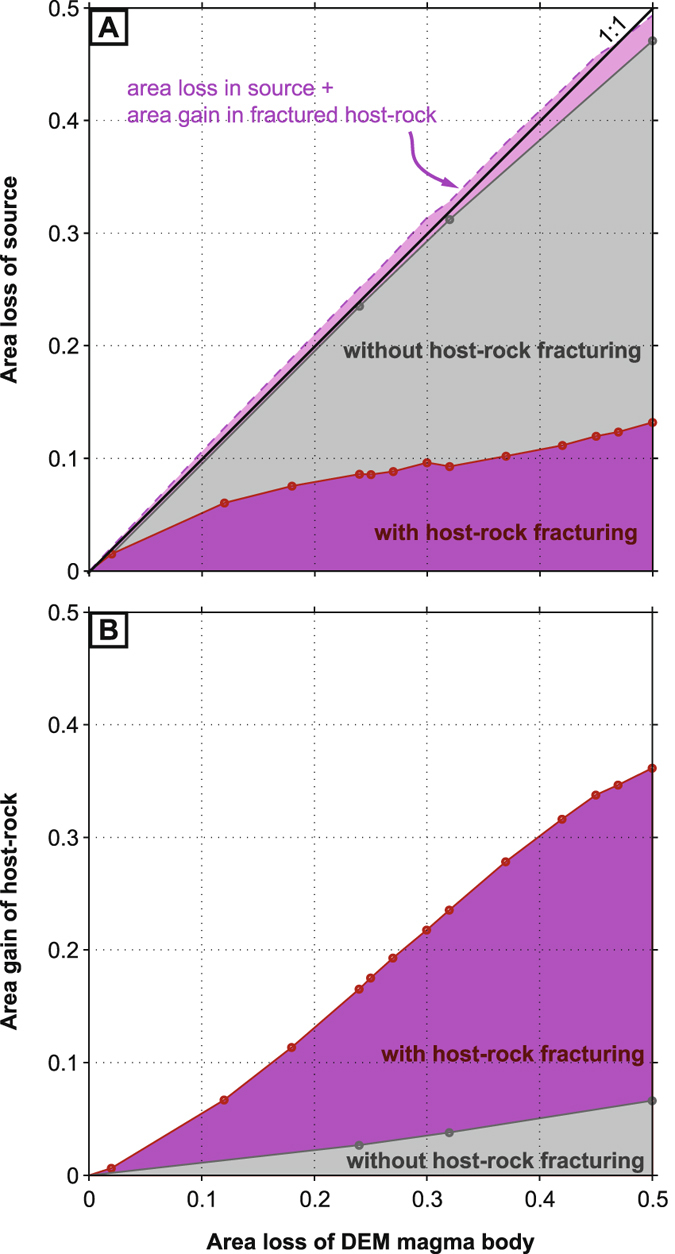
Effect of host-rock fracturing on area changes in the DEM model and in the elastic-deformation source. (**A**) Area loss in the two-plane elastic-deformation source vs area loss in the DEM magma reservoir. All values are normalized to the initial area of the DEM magma body. (**B**) Area gain in the DEM host-rock vs area loss in the DEM magma reservoir. Both plots show values for scenarios in which host-rock fracturing was either permitted or prohibited. In addition, plot (A) shows the sum of the absolute area lost by the elastic-deformation source plus the area gained by the host-rock in scenarios with host-rock fracturing permitted. Note that this sum closely matches the ‘true’ area loss of the DEM magma body.

Figure 6 shows that the apparent area loss associated with the elastic-deformation source is an underestimate of the true area loss of the DEM magma body when fracturing is permitted. In contrast, the apparent and true area losses closely match when fracturing is not permitted (values plot almost on a 1:1 line in Fig. 6A **)**. Due to reservoir depletion, the DEM host-rock undergoes dilation that is far more pronounced with host-rock fracturing than without (Fig. 6B **)**. Moreover, the true area loss of the DEM magma body is almost equal to the sum of the absolute area loss of the elastic-deformation source plus the area gain of the fractured host-rock **(**values plot almost on the 1:1 line in Fig. 6A **)**. This shows that this mismatch between the ‘apparent’ and ‘true’ magma depletion is directly related to the dilation of the host-rock associated with fracturing.

Finally, results for other initial geometries of the DEM model are given in the Supplementary Figure S9. These show that, prior to surface collapse, the effects of host-rock fracturing on the elastic-deformation source become less pronounced as the depth/diameter ratio of the DEM magma body decreases (i.e. for a shallower magma body). For a depth/diameter ratio < 1, the source depth more closely reflects the true depth prior to surface collapse. This is because the host-rock above a shallower magma body fails through to surface at a smaller depletion^26, 48^, and so inelastic strains due to subsurface fracturing are smaller before the onset of surface collapse. For a depth/diameter ratio > 1.5, however, the effect of fracturing on the elastic-deformation source is significant even though fracturing is not observed at the surface. The depth/diameter ratio at which such non-elastic (i.e. fracture-related) effects become substantial is likely to also depend on mechanical factors such as Young’s modulus, Poisson’s ratio and rock strength, but their exploration is beyond the scope of the present work.

## Discussion and Conclusions

The changes in shape and depth of the elastic-deformation source resolved for our DEM models closely match those reported for the Mar-April 2007 eruption of Piton de la Fournaise (Fig. 5). For a given source potency (strength), such changes are sensitive to two interacting factors: (1) the displacement profile shape and (2) the horizontal/vertical (H/V) displacement ratio^3, 4, 7, 11^. Displacement profiles that decay more sharply from their maxima or minima favour shallower and/or more-sill-like elastic-deformation sources. Higher H/V values favour stock-like sources (ideally H_max_/V_max_ > 0.4), whereas lower H/V ratios favour sill-like sources (ideally H_max_/V_max_ < 0.4). The DEM displacement profiles progressively sharpen as strain localises onto upward-migrating fracture systems in the host-rock above the magma body **(**Fig. 4 **)**. Up to the point of surface collapse, both the sharpening profiles and the increased H/V ratio account for the elastic-deformation source’s progressive upward movement and its shape change from sill-like to stock-like. After surface collapse, the H/V values decline but remain high, and so the return to a sill-like shape is mainly due to the markedly-increased sharpness of the displacement profiles. Significantly, the H/V ratio at Piton de la Fournaise^30^ also generally increased during the syn-eruptive deflation stage on April 2^nd^–5^th^, then decreased during the collapse stage on April 5^th^–6^th^ (Fig. 1B). Cumulative post-collapse displacements^45^ decayed sharply from the caldera rim, with H_max_/V_max_ ~ 1.8.

Our study therefore supports the hypothesis that such enigmatic changes in the elastic-deformation source at Piton de la Fournaise were primarily a result of inelastic host-rock deformation that migrated upward from the magma reservoir and culminated in caldera collapse at the surface^30^. The host-rock above the magma body likely underwent an initial phase of incremental sub-surface fracturing from April 2^nd^–5^th^, followed perhaps by a more coherent piston-like collapse style on April 5^th^–6^th^ once a through-going ring fault system had developed. Our interpretation is supported by the increased number and upward migration of earthquakes below the volcano summit^34, 36^. A very similar development of collapse at Miyakejima volcano in 2000 is indicated by recent analysis of seismicity there^49^. Collapse style at Piton de la Fournaise was therefore structurally more complex than a previously-assumed simple piston^37, 45^, probably because of the high depth/diameter ratio of the magma body^25, 26, 50^.

**Figure 7:**
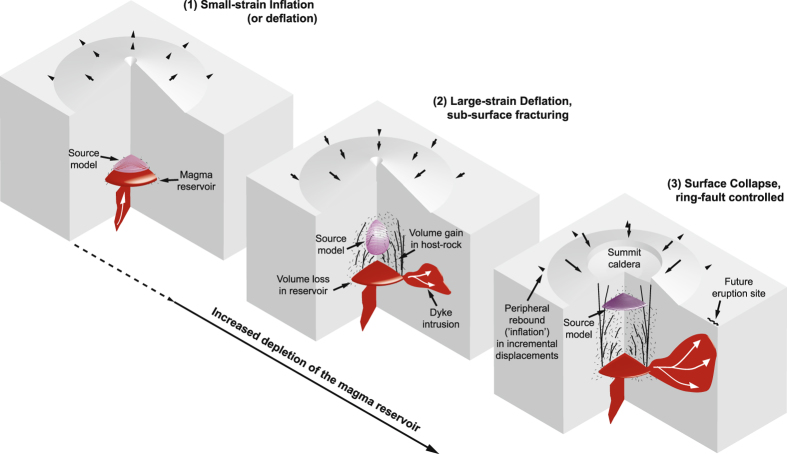
Schematic summary of the influence of host-rock fracturing on elastic-deformation source models of volcano deflation. See the main text for further details. In the scenario depicted here, the onset of non-elastic deformation occurs prior to the onset of eruption^29^. Fracturing leads to a volume gain in the host-rock, which at least partly accounts for mismatch between erupted/intruded volumes and elastic-deformation source volume estimates. Our study shows that upward migration of a source model during volcanic unrest may be solely fracture-induced and hence may not necessarily be indicative of an impending eruption at the volcano summit.

We conclude that where lines of evidence (e.g. seismicity, field observations) exist to indicate substantial host-rock fracturing during volcano deflation, source models assuming linear elasticity should be interpreted to reflect not only the influence of a magma body, but also a zone of inelastic host-rock deformation around and/or above the body (Fig. 7). A similar conclusion was reached for volcano inflation at Kilauea, Hawaii^4, 11^, where displacements are best fit by a vertically-elongated ellipsoidal source. This has been interpreted as a set of vertically-stacked magma bodies or as a ‘pseudo-chamber’ comprised of a combination of magma bodies plus an envelope of fractured host-rock. Several elastic-deformation sources reported for other volcanoes in the literature comprise gently-inclined (4–25°) planes^51–53^ or gently-plunging prolate ellipsoids^52, 54^. The geological meaning of such inclination is often uncertain, although it is commonly linked with the shape and orientation of subterranean magma bodies^51, 53, 54^. Although no surface discontinuities were observed in those cases, our models show that inclination of an elastic-deformation source may result above a non-inclined magma body from uneven or ‘asymmetric’ development of host-rock fracturing in the sub-surface. The unevenness or asymmetry arises in our DEM models, and in nature, because fracture system development is affected by local heterogeneities.

In common with past studies on volcano inflation at Hawaii^4^, Rabaul^55^ and at Campi Flegrei^19^, we conclude that, with substantial fracture-related host-rock deformation, a source depth derived from elastic modelling may considerably underestimate the magma body depth (see also^30^). We further show that the sub-surface development of host-rock fracturing leads to upward migration of an elastic-deformation source, and upon surface collapse, causes a relaxation or rebound of peripheral displacements (Figs 1A and 7). Such consequences may be misinterpreted as new intrusion and/or ascent of magma^37^.

Our study also indicates that substantial host-rock fracturing can lead to source volume changes that considerably underestimate the true magma volume change (Fig. 6). Erupted lava volume at Piton de la Fournaise in 2007 was ~150 × 10^6^ m^3^ DRE^38^. The intruded magma volume was perhaps ~20 × 10^6^ m^3^ ^35^. The collapse volume^34^ was ~100 × 10^6^ m^3^. This gives a ratio of collapse volume to extracted magma volume of ~0.6. The ratio of collapse area to reservoir area loss for the DEM model is ~0.3 at 50% depletion (Fig. 6), but this increases to ~0.5 at 90% depletion. Moreover, the estimated erupted volumes for April 2^nd^–5^th^ and April 5^th^–7^th^ are about 30 × 10^6^ m^3^ DRE and 50 × 10^6^ m^3^ DRE, respectively^35^, whereas volume changes estimated by the elastic-deformation sources for these periods are 1.00+/−0.3 × 10^6^ m^3^ and 1.46+/−0.5 × 10^6^ m^3^, respectively^30^. Thus, the elastic-deformation source model underestimated the April 2007 magma body’s volume change by at least a factor of 20 to 50. These mismatches between the volumes of the caldera, the elastic-deformation source and the erupted/intruded magma may relate in part to effects of host-rock compliance and magma compressibility^56^, but our study indicates that these discrepancies can also be partly attributed to dilation due to host-rock fracturing (Fig. 6). Occurrence of such dilation at Piton de la Fournaise in April 2007 is supported by decreases in both seismic velocity^57^ and gravitational acceleration^58^ in the summit area.

There are several other natural or anthropogenic processes on Earth with structural and surficial similarities to volcano deflation^59, 60^, such as mine collapse^61^, sinkhole development^62^ and subsidence induced by underground nuclear tests^63, 64^. One consideration of non-elastic strains on elastic-deformation source models for a geodetically-observed mine collapse came to conclusions similar to those here^61^. We therefore anticipate that the findings of our study and the future use of the DEM will help to better understand geodetic and seismic observations of high strain subsidence phenomena in general.

## Methods

The set-up for Distinct Element Method simulations of magma-body deflation is shown in Supplementary Figure S1. The simulations are run with Itasca Consulting Group’s DEM software Particle Flow Code in Two Dimensions (PFC2D). Each model comprises a gravitationally-loaded assemblage of rigid disc-shaped particles contained within three rigid boundary walls^26, 33, 48, 65^. The radii of the randomly-emplaced particles are uniformly distributed between 10 and 6 m. The particles interact with each other and with the boundary walls via a linear force-displacement law with Coulomb friction. Particle-wall contacts are cohesion-less and friction-less. From a convergence test of elastic surface displacement against DEM model size (Supplementary Figure S2), and to achieve a reasonable computation time for multiple realisations of each model geometry, we adopt assemblage dimensions of 5 × 15 km for the main model set.

Host-rock is represented in the DEM simulations by bonded particles with a contact friction coefficient of 0.5. The beam-like inter-particle bonds (‘parallel bonds’) are elastic and break if their tensile or shear strength is exceeded. As broken bonds accumulate, strain can localise and large-displacement fracture systems can develop. Consequently, the bonded particle assemblage undergoes a transition from elastic quasi-continuum behaviour to inelastic discontinuum behaviour. Simulated rock mechanics tests^26^ show that the host-rock’s bulk material properties include a bulk density of 2400 kgm^-3^, a Poisson’s ratio of 0.17–0.25, a Young’s modulus of c. 5 GPa, an internal friction coefficient of 0.56, an unconfined compressive strength of c. 10 MPa and a tensile strength of c. 3 MPa. The effect of gravity on model behaviour is calibrated by running simulated rock mechanics tests in which confining pressure is systematically increased^26, 33^. The consequences for the bulk material’s behaviour are that: (1) Young’s modulus increases slightly with depth; (2) Poisson’s ratio decreases slightly with depth; (3) failure mode changes from tensile near the surface to shear at depth; and (4) peak host-rock strength increases with depth^26, 48^. Such mechanical responses are expected in nature for upper-crustal volcanic rock masses at a large (>100 m) scales^66^.

The magma body is represented as a zone of non-bonded particles with a contact friction of 0.01. The magma body is sill-like with dimensions of 1200 m × 300 m. Eruption-related deflation is simulated by incrementally reducing the area of each magma body particle by a constant value in each time step, sufficiently small to achieve quasi-static conditions. We hence assume lateral magma outflow, perpendicular to our 2D model, as occurred during the March-April 2007 activity at Piton de la Fournaise^34^. To calculate the area gain of the host rock in Fig. 6, we simply subtract the area of subsidence at the model surface from the area loss of the reservoir. For further description and discussion of the DEM models, see references^26, 48^.

We blindly compare surface displacements of the DEM models to those predicted by an analytical solution for deformation related to rectangular dislocations in a linearly-elastic, homogeneous, isotropic half-space^6^. For this step, we use a Young’s modulus of 5 GPa and a Poisson’s ratio of 0.25. Our preferred deformation source comprises two dislocations that intersect perpendicularly at their mid-points. Since the 2D-DEM models have no out-of-plane stresses and strains, we approximate these conditions in the analytical solution by: (1) setting the dislocation lengths to 1000 km, (2) setting the dislocations to lie normal to a plane of observation midway along their length, and (3) considering surface displacements from the plane of observation only. Parameters free to change were: (a) the position of the dislocations’ intersection (i.e. source’s center), (b) the width of each dislocation, (c) the dip of the dislocations (though fixed with respect to the other) and (d) the displacement normal to each dislocation (i.e. ‘opening’ or ‘closing’).

An elastic-deformation source composed of three mutually-orthogonal dislocations with uniform displacement normal to them is termed a compound dislocation model (CDM)^47^. For large ratios of centre depth, d, to semi-major axis, a, (d/a > ~2), a CDM produces a displacement field equivalent to that of an infinitesimally-small pressurized cavity (spherical or triaxial point source), as simulated by a centre of dilatation or by three mutually-orthogonal force dipoles^4, 22, 47^. For smaller d/a ratios (1 < d/a < ~2), a CDM can also serve as equivalent to a cavity of finite size (a ‘volumetric source’)^47^. For the purpose of satisfying two-dimensional conditions, as outlined above, we treat the displacement on one of the three dislocations as negligible and adopt a two-dislocation CDM instead. The two-dislocation source can therefore be considered to represent a pressurised, prolate or tri-axial, ellipsoidal cavity.

To help visualize how the two-dislocation source might relate to the shape of an enclosed sub-surface body (i.e. a volumetric source, such as an ellipsoid), we define a ‘strength ellipse’. The product of width and closing on each dislocation represents an area change. This is linked to the strength, or potency, of each plane in the source, and it is proportional to the geodetic moment. To show the relative sizes of the area changes on each plane, the strength ellipse’s major axis is given an arbitrary length and is set parallel to the plane with the larger area change. The ellipse’s minor axis is then scaled by the ratio of the smaller to the larger area change.

Given the finite vs. infinite boundary positions in the DEM and analytical models, respectively, a mismatch between the DEM and analytical surface displacements is typically observed in the far-field. Consequently, we compare the analytically-predicted displacements to those within the central 12 km of the DEM model, where convergence tests show only small changes in the elastic horizontal and vertical displacement components (<8% and <1%, respectively) from increasing the DEM model size (Supplementary Figure S2). We also verify that enlarging the DEM model does not greatly change the analytical model results based on this sampling of the central 12 km (Supplementary Figure S3). Finally, we exclude the possibility of significant boundary influences on the main effects reported here by conducting the same elastic-deformation source optimisation for DEM models in which host-rock fracturing was prohibited (see Supplementary Figure S4). To prohibit fracturing, we set the inter-particle bond strengths to 1000 MPa. The results of this test demonstrate that the changes in the elastic - deformation source are only a consequence of host-rock fracturing.

We exclude displacements from the central part of the DEM model surface that are affected by large collapse-related fractures (shaded areas in Fig. 3). If one had displacement data in this area in nature, one would alter the simple source modelling scheme used here to take the fractures into account (e.g.^32^). Data are commonly unavailable in the central area, however, especially in the case of a large caldera collapse (e.g.^28, 30^), as at Piton de la Fournaise.

Errors in the analytical solution may become significant at d/a < 1–2^4, 47^. The optimum two-dislocation source resolved here has d/a > 2 prior to surface collapse, but d/a < 1 after surface collapse, particularly for incrementally modelled displacements (Fig. 2 and Supplementary Figure S7). Especially for the post-surface-collapse stage, one might therefore favour the use of shallow finite source geometries with a continuum-based numerical approach at the cost of greater computational intensity. Nonetheless, as shown by^30^, we anticipate that processes simulated here will produce similar overall patterns of change for a simple numerically-modelled elastic-deformation source.

An optimum analytical source model whose surface displacements best explain the DEM model displacements is found through a Monte-Carlo-type (evolutionary) optimization of possible source configurations arising from the free parameters^67, 68^. Weighting of the DEM-to-analytical model fit is uniform. The stability of each optimum analytical model result is checked by a Markov-chain Monte Carlo sensitivity analysis^67^. This produces a likelihood distribution for each source parameter and enables the identification of parameter trade-offs (Supplementary Figure S6).

A single dislocation, which is a commonly-used elastic-deformation source, was also considered in our inversions. Note that the same general patterns of tilting and upward migration are seen also for the single dislocation source (Supplementary Figure S5), and thus these patterns are not strongly related to the choice of source. We opted for the two-dislocation source, because it consistently yields a better fit to the DEM data, especially where fracturing is significant (compare Supplementary Figures S5 and S8). Other commonly used elastic-deformation sources, such as infinitesimal^1^ or finite^69^ spherical cavities, prolate ellipsoids^5^, or penny-shaped cracks^7^, are axisymmetric about a vertical axis and thus unsuitable here given the 2D nature of the DEM model. This limitation may be overcome in future as increasing computational capacity makes an axisymmetric or a fully 3D-DEM approach viable.

### Data availability

The datasets generated during and/or analysed during the current study are available from the corresponding authors on reasonable request.

## Electronic supplementary material

Supplementary Text and Figures
