# Supplementary material for: Effects of Host-rock Fracturing on Elastic-deformation Source Models of Volcano Deflation
Source: Sci Rep. 2017 Sep 8;7:10970. doi: 10.1038/s41598-017-10009-6 (PMC5591261; doi:10.1038/s41598-017-10009-6)
Supplement: Supplementary file 1 — Supplementary Text and Figures [file 41598_2017_10009_MOESM1_ESM.pdf]

Supplementary Text and Figures to

"Effects of Host-rock Fracturing on Elastic-deformation Source Models of Volcano Deflation"

Eoghan P. Holohan, Henriette Sudhaus, Thomas R. Walter, Martin P.J. Schöpfer, & John J. Walsh

1) Combining and testing the DEM and analytical approaches

The configurations of the two modelling approaches used in this study are shown in **Figure S1**. To combine the modelling approaches robustly and to exclude artefacts form the different boundary conditions of each approach, we ran several tests.

Firstly, we performed a convergence test of low strain (elastic) surface displacement in the DEM model against the DEM model size (**Figure S2**). From this, and to achieve a reasonable computation time, we adopted assemblage dimensions of 5 x 15 km for the model in the main text. A close comparison of displacements for elastic and non-elastic deformations from this model and the next largest tested (5 x 25 km) shows that the difference between normalized displacements within the inner 12 km of the DEM is less than 15% for horizontal component and 1 % for the vertical (**Figure S3**). Moreover, there is no systematic increase in these differences with increased depletion. Consequently, the effect of increasing the DEM model size is small – only slightly decreasing the optimum and maximum likelihood of the source depth by ca. 100 m (i.e. by less than 5% of the true depth).

Secondly, we tested if changes in the elastic-deformation source attributes, especially depth, could be explained as a consequence of an interaction between increased reservoir depletion and a boundary effect, rather than fracturing of the DEM material. To do so, we ran DEM models in which fracturing was prohibited by setting elastic bond strengths to extremely high values (1000 MPa). Moreover, the reservoir depletions and absolute 'closing' values were set to be the same as in the fractured model in the main text. The forced 'closing' of the DEM magma reservoir under these

elastic conditions was achieved by simultaneously shrinking the reservoir particles and assigning a constant velocity to the particles bounding the reservoir. The velocity assigned to each boundary particle was the value attained by that particle at the end of an initial gravitationally-driven closing stage of up to 2% depletion. This test shows that without fracturing the optimum elastic source model characteristics are largely unaffected (**Figure S4**) – see also **Figure 5** in the main text. The depth and orientation of the elastic-deformation source, as well as the shape of the associated strength ellipse, are relatively stable as reservoir depletion increases. Only the area change associated with the elastic-deformation source increases, and does so linearly with respect to reservoir depletion in the DEM model (see **Figure 6a** in the main text). By so isolating the effect of fracturing, this test demonstrates that the changes in the source attributes described in the main text are unequivocally due to host-rock fracturing.

2) Alternative elastic-deformation sources

As noted in the **Methods** section of the main article, a one-plane elastic-deformation source performs roughly as well as the two-plane source for the low-strain phase of deformation in the DEM model, but consistently performs more poorly than the two-plane source during the higher strain (inelastic) phases (**Figure S5**). For cumulative displacements, RMS errors for the optimum one-plane and two-plane elastic-deformation sources are 0.002 vs 0.0.002, 0.172 vs 0.023 and 0.471 vs 0.042, at 2%, 32% and 50% depletion, respectively (compare **Figures S5 and S8**). The more complex two-plane deformation source was therefore preferred. Nonetheless, note that the one-plane source also migrates upward and tilts as a result of host-rock fracturing (**Figure S5**).

Other commonly-used analytical solutions for deformation sources either are inappropriate for the 2D conditions of the DEM model or limit the cross-sectional shape of the source to a circle. See the **Methods** section of the main article for further discussion of these limitations and of the relationship of the two-plane source to other elastic-deformation source solutions.

3) Parameter trade-offs in the elastic-deformation source

Sensitivity analysis for the two-plane elastic-deformation source reveals trade-offs between several parameters of the source. **Figure S6** shows sensitivity analysis results for modelling cumulative displacements of the DEM model in the main text arising from depletions of 2%, 24% and 32%. The likelihood distributions of source depth, x-position, dip and moment form well-defined Gaussian distributions, whereas those of width and closing show skewed distributions with long tails toward larger values. The bivariate plots show clear correlation between closing and width on both planes. In detail, closing increases as width decreases. This indicates a strong trade-off between closing and width, such that neither can be independently constrained. The product of closing and width, i.e. the area change, in the two-plane source model is more stable and easier to interpret, however. While the total area change increases monotonically with reservoir depletion, the ratio between the area change on plane A versus that on plane B decreases. We visualize this as the 'strength ellipse' of the two-plane source model, which here develops from 'sill-like' characteristics to 'stock-like' characteristics and vice-versa (see also the Methods section of the main article).

4) Modelling of incremental vs cumulative displacements

Figures S7 and S8 show expanded results for modelling of cumulative displacements. These should be compared with the results for modelling of incremental displacements, as shown in **Figures 2 and 3**. As noted in the main article, the chief difference between these results is that the upward migration, tilting and shape change of the elastic-deformation source is less pronounced in the cumulative case, as the summing of earlier displacement dampens the impact of later displacement changes. These figures nonetheless also show that cumulative modelling of the DEM displacements produces the same general pattern of change in the elastic-deformation source.

5) Effect of the DEM magma reservoir's depth/diameter ratio

The greater the depth/diameter ratio of the DEM magma reservoir, the more significant the effects of host-rock fracturing will be on the analytical elastic-deformation source prior to host-rock

fracturing reaching the surface **(Figure S9)**. For a 1200 m deep magma body, the optimum elastic-deformation source depth appears to be slightly less than the true depth, but the likelihood distribution indicates that this is insignificant. For the 1800 m deep magma body, however, the upward movement of the optimum source is significant. For the 2400 m deep model shown in the main article, the effect of sub-surface fracturing is yet more pronounced. Note also here in **Figure S9** that at 2% depletion (i.e. in the fully elastic stage of DEM model behaviour), the true depths of these shallower magma bodies are more precisely recovered by the elastic-deformation source than is the case for the deeper magma body shown the in main article. This is probably because the two-plane source is a compound dislocation model, which for shallow depths can serve well as equivalent to sources of finite geometry (see **Methods** section of main text for discussion).

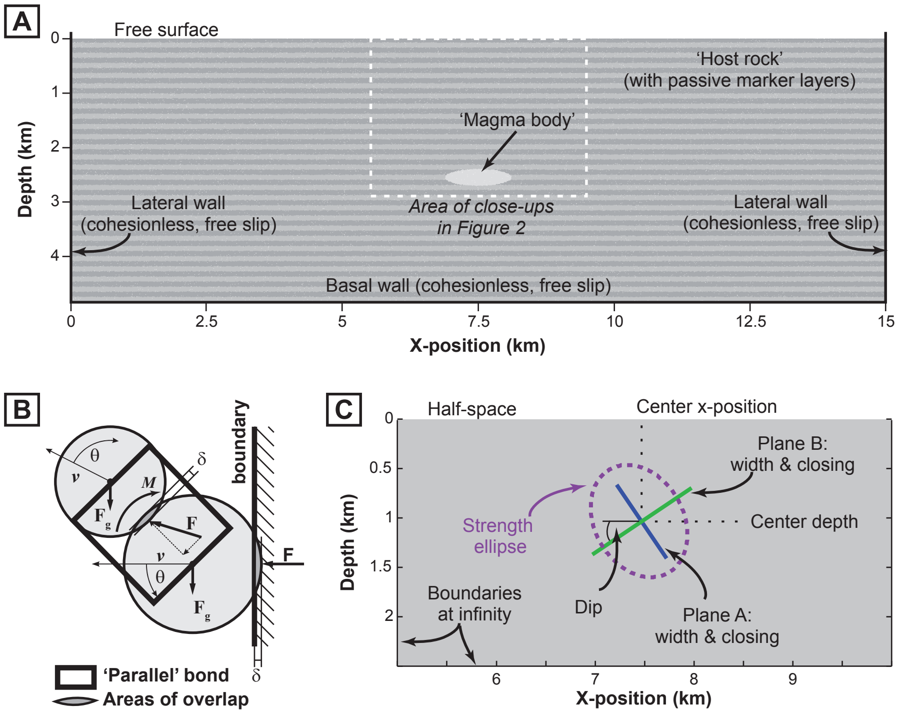

Figure S1: Overview of the DEM and elastic-deformation source model set-ups. **(A)** The 2D-DEM model prior to magma chamber deflation; **(B)** Sketch of the disk-like particles, beam-like 'parallel' bonds and rigid boundary walls that make up the DEM model. This also depicts the forces (F), which arise through gravitational acceleration (F_g), and spring-like reactions at particle-particle or particle-boundary overlaps (δ), as well as the related moments (M), rotations (θ) and velocities (v). **(C)** Cross-section sketch of the deformation source model, which comprises two rectangular dislocation planes in a homogeneous, linearly elastic half-space. To approximate the 2D conditions of the DEM models, the dislocation planes are fixed to be perpendicular to each other and to the cross-sectional plane. They are also fixed to be 1000 km long in the direction normal to the cross-sectional plane. Surface displacement data to be compared to the DEM model are taken from along the cross-section only. Parameters labelled in black are free to change in the source modelling. By convention, the more steeply-dipping plane is coloured blue and labelled Plane A. The strength ellipse represents the normalized product of width and closing on each plane (see Methods for details).

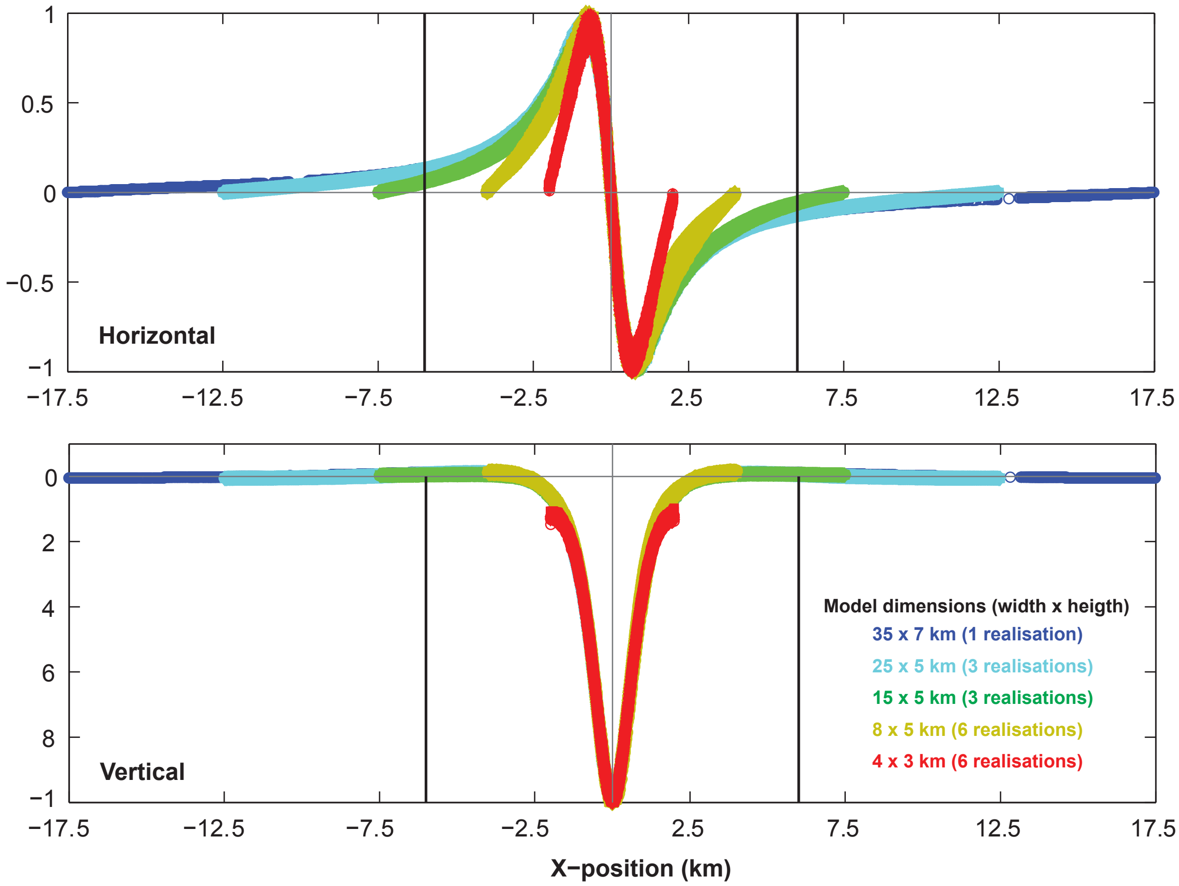

Figure S2: Convergence test results for 1% depletion of a 750 x 500 m elliptical DEM magma body at a depth of 750 m with varying boundary wall dimensions. Shown are the horizontal (upper plot) and vertical (lower plot) components of displacement, each normalized to the maximum of that component. Within the area used for the elastic-deformation source modelling (vertical black lines), the difference between the 5 x 15 km and the 5 x 25 km models is less than 10% of the maximum displacement in the horizontal component and less than 1% in the vertical component.

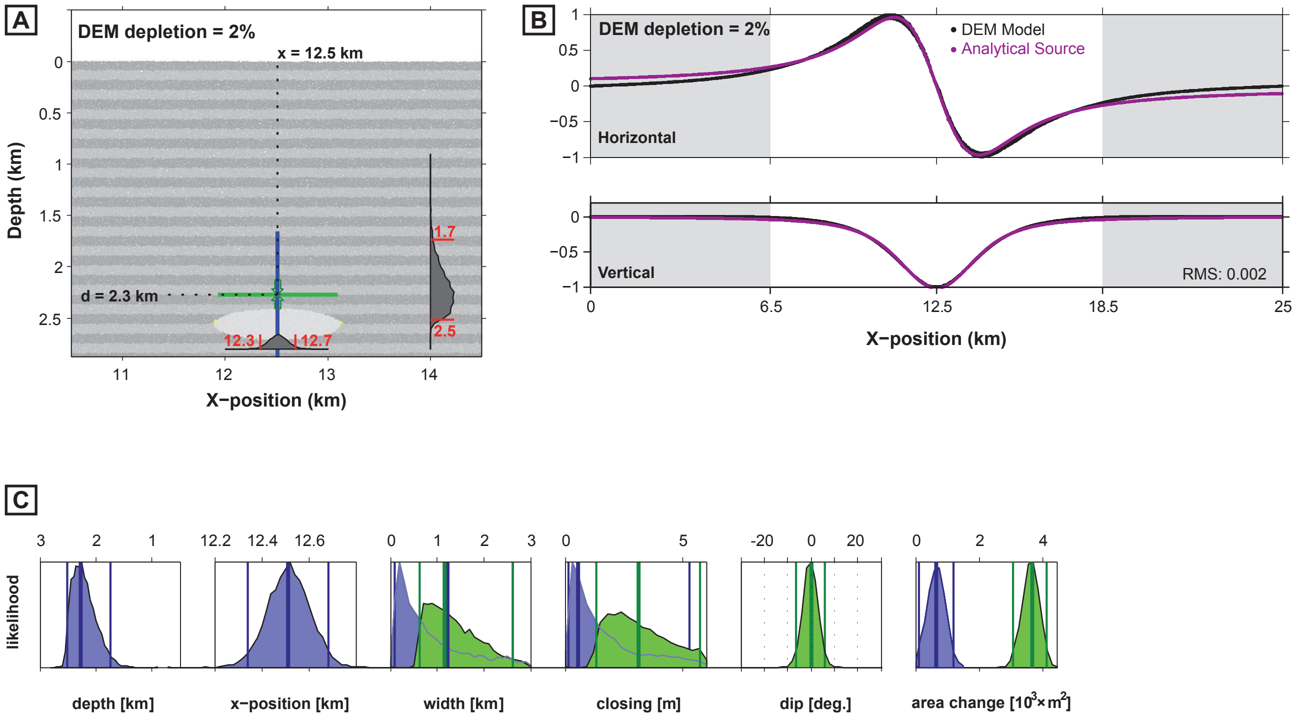

Figure S3: Effect of increased DEM model size on the elastic-deformation source results. Shown here are source modelling results for displacements induced by 2% depletion of a DEM magma body within a particle assembly with boundary dimensions of 5 x 25 km. **(A)** Close-up view of the DEM model and the optimum source model. **(B)** The normalized horizontal (top) and vertical (bottom) components of surface displacement. Displacements are normalized to the maximum of that component. The grey shaded areas denote parts of the DEM surface displacement profiles that were excluded from elastic source modelling (see main text for details). The misfit of the elastic-deformation source's displacements to those of the DEM model is indicated by the normalized root mean squared (RMS) error. **(C)** Likelihood distributions for the source parameters. The sixth plot shows the area change on each source plane, i.e., the product of each plane's width and the closing across it. The thick blue and green lines correspond to the optimum values for source planes A and B respectively, while the fine blue and green lines correspond to the 2.5 and 97.5 percentiles.

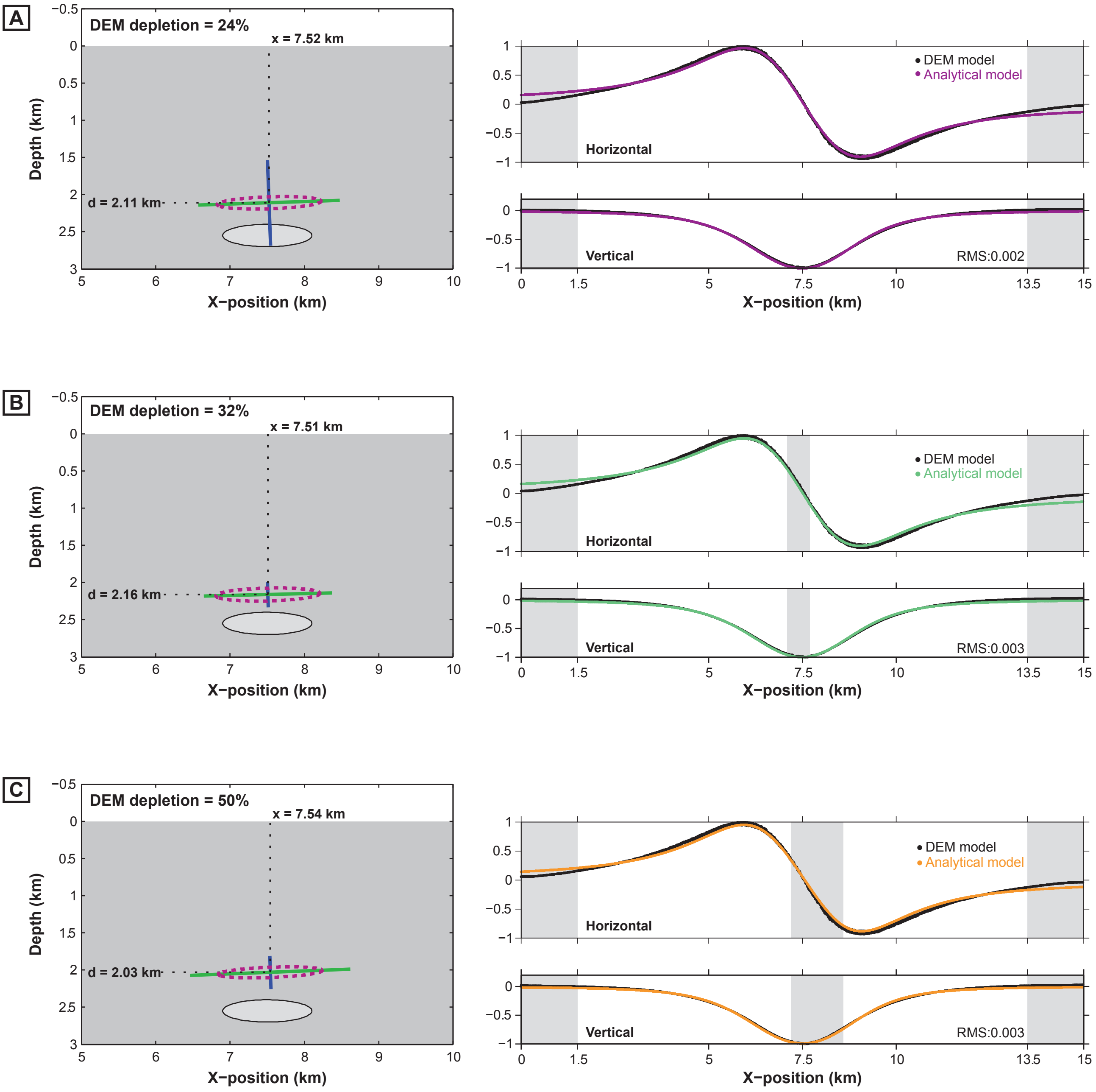

Figure S4: Source modelling results for deflation without host-rock fracturing. **(A)** 24% depletion, with DEM magma body closing of 65 m. **(B)** 32% depletion, with closing of 89 m. **(C)** 50% depletion, with closing of 144 m. Reservoir closing at each stage is equivalent to that in the model with host-fracturing (see Figures S7 and S8). Shown on the left for each stage is a view of the optimum elastic-deformation source with respect to the pre-deflation outline of the DEM magma body. Shown on the right are the normalized horizontal (top) and vertical (bottom) components of cumulative surface displacement. Displacements are normalized to the maximum of each component. The grey shaded areas denote parts of the DEM surface displacement profiles that were excluded from elastic-deformation source modelling (see main text for details). The misfit of the elastic-deformation source displacements to those of the DEM models is indicated by the normalized root mean squared (RMS) error. In the absence of host rock fracturing, the elastic source's position, its strength ellipse and the fit of its displacements are essentially unchanged with increased depletion.

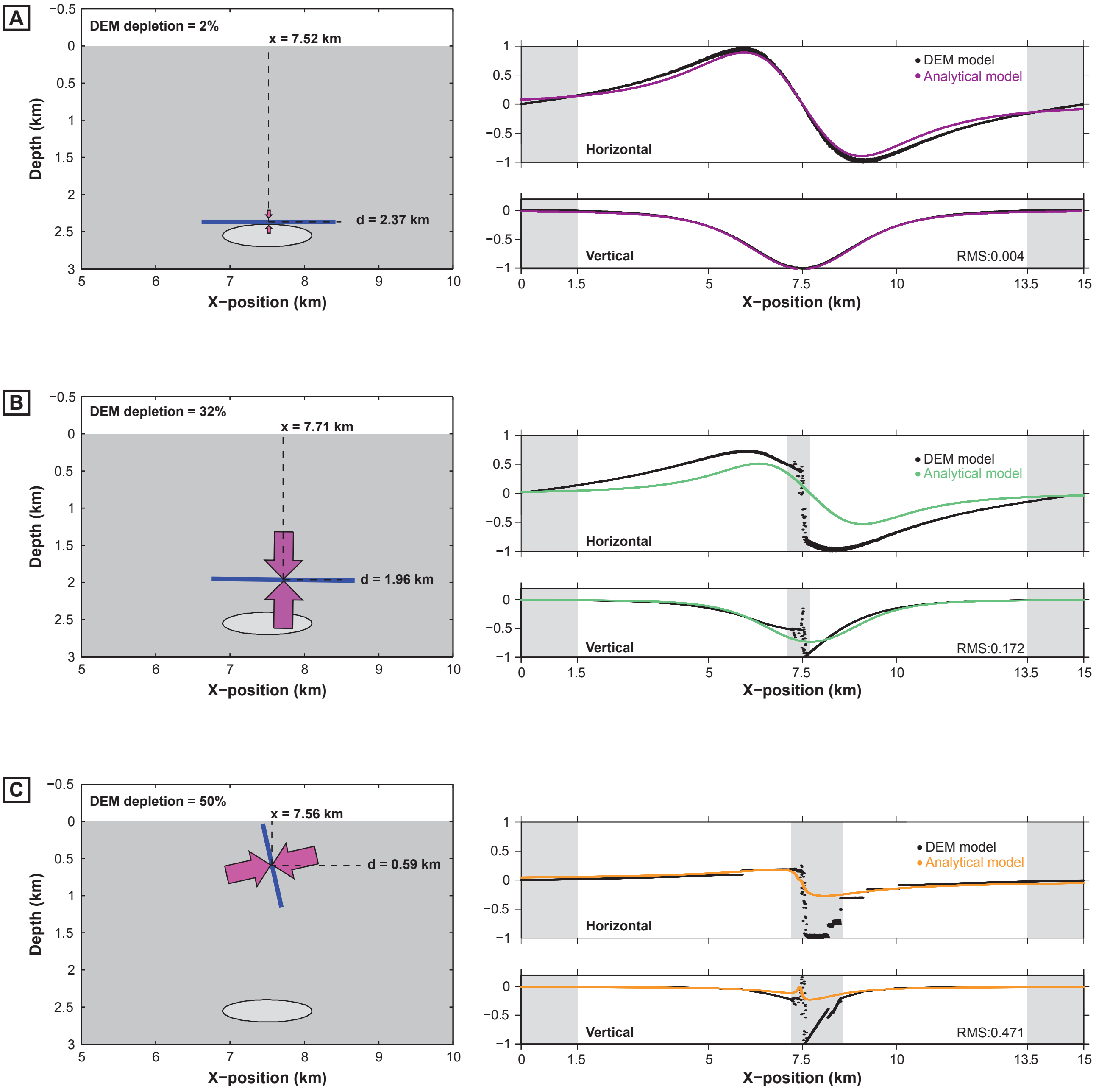

Figure S5: Single-dislocation source modelling results for cumulative displacements. To the left are views of the optimum elastic-deformation sources with an outline of initial DEM magma body at depletions of **(A)** 2%, **(B)** 32% and **(C)** 50%. The size of the pink arrows is scaled to the magnitude of closing across the dislocation. To the right are the corresponding horizontal (top) and vertical (bottom) components of surface displacement. Displacements are normalized to the maximum of that component. The grey shaded areas denote parts of the DEM surface displacement profiles that were excluded from source modelling (see Methods for details). The misfit of the source model displacements to those of the DEM models is indicated by the normalized root mean squared (RMS) error. Note that this misfit is consistently greater than that of the two-plane source (Figures 3 and S7).

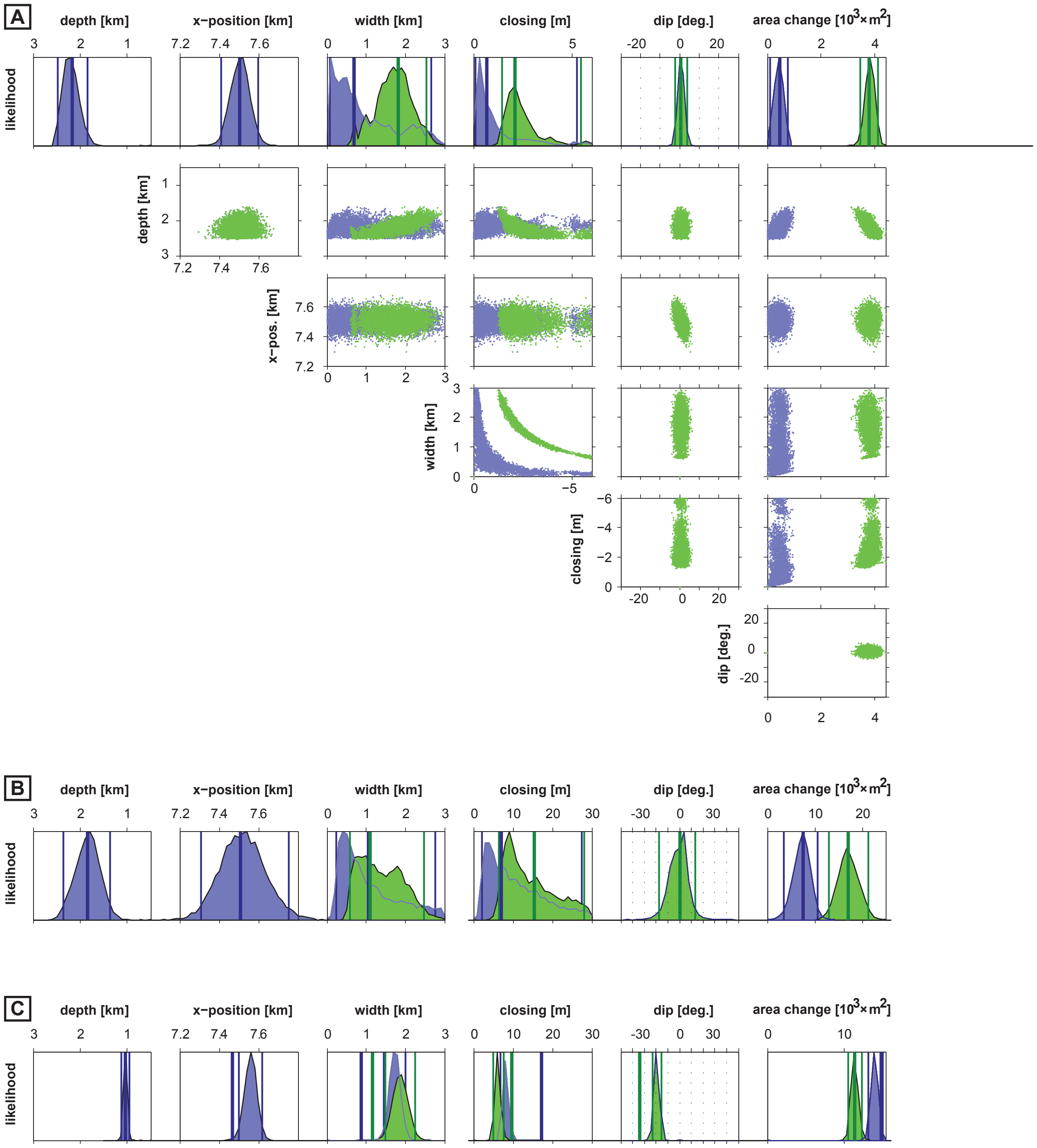

Figure S6: Sensitivity analysis of source model results for cumulative displacements. Shown are likelihood distributions for the two-plane source's parameters at DEM magma body depletions of **(A)** 2%, **(B)** 24%, and **(C)** 32%. The sixth likelihood distribution plot shows the area change associated with each plane, i.e., the product of each source plane's width and the closing across it. The thick blue and green lines correspond to the optimum values for source planes A and B respectively, while the fine blue and green lines correspond to the 2.5 and 97.5 percentiles. For the 2% depletion results in (A), bivariate plots are also shown for each of the two-plane source's parameters to illustrate trade-offs between them. The bivariate plots for depletions of 24% and 32% show patterns very similar to those for 2% depletion.

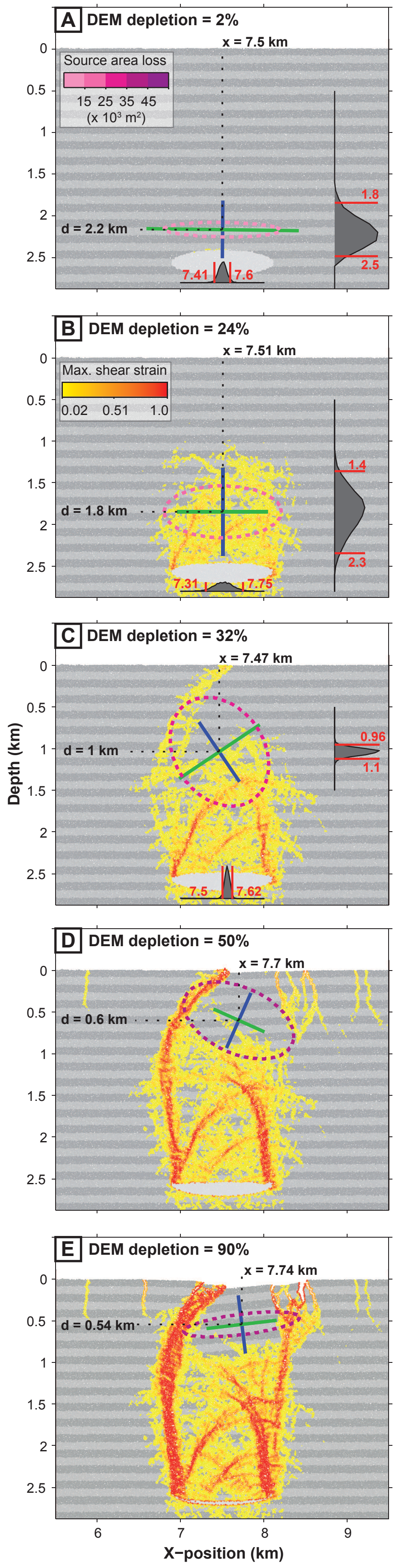

Figure S7: Effects of non-elastic host-rock deformation on elastic-deformation sources for cumulative displacement. **(A)** Low strain (elastic) deformation in the DEM model. **(B)** Fracturing in the subsurface at higher strains. Inelastic deformation is shown by colouring each particle according to the maximum finite shear strain around it. **(C)** Inelastic deformation becomes markedly 'asymmetric' and reaches the surface. **(D-E)** Surface collapse and development of through-going fracture systems. Note that the changes in the source depth and the strength ellipse are more pronounced when displacements are modelled incrementally, as in the case of Piton de la Fournaise - see Figure 2 and main text. Note also the changes in tilt direction due to the generation of new fractures as deformation progresses.

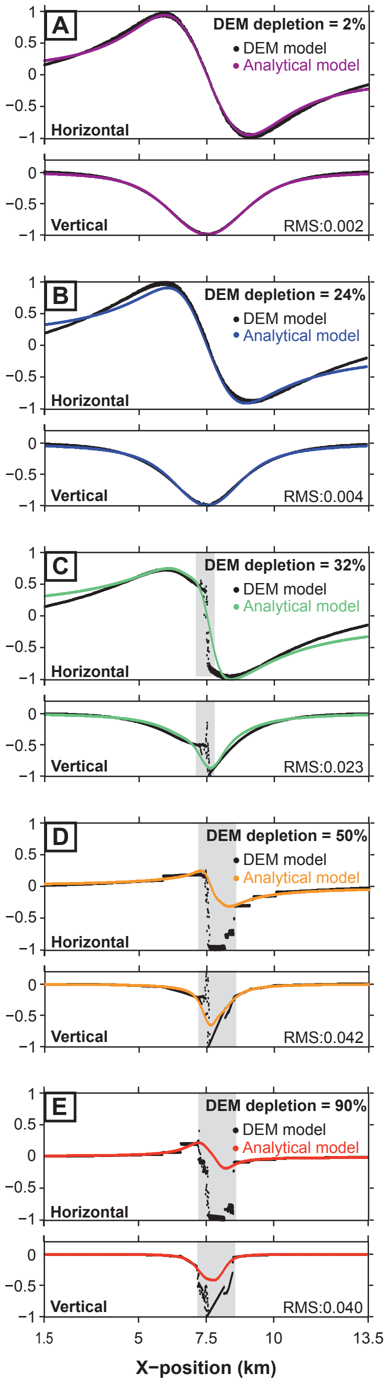

Figure S8: Cumulative surface displacement profiles from the DEM model and the optimum elastic-deformation source. The horizontal (top) and vertical (bottom) components are plotted for DEM reservoir depletion stages of: **(A)** 2%, **(B)** 24%, **(C)** 32%, **(D)** 50% and **(E)** 90%. Displacements are normalized to the maximum of that component. See the caption to Figure 3 and the main text for further details.

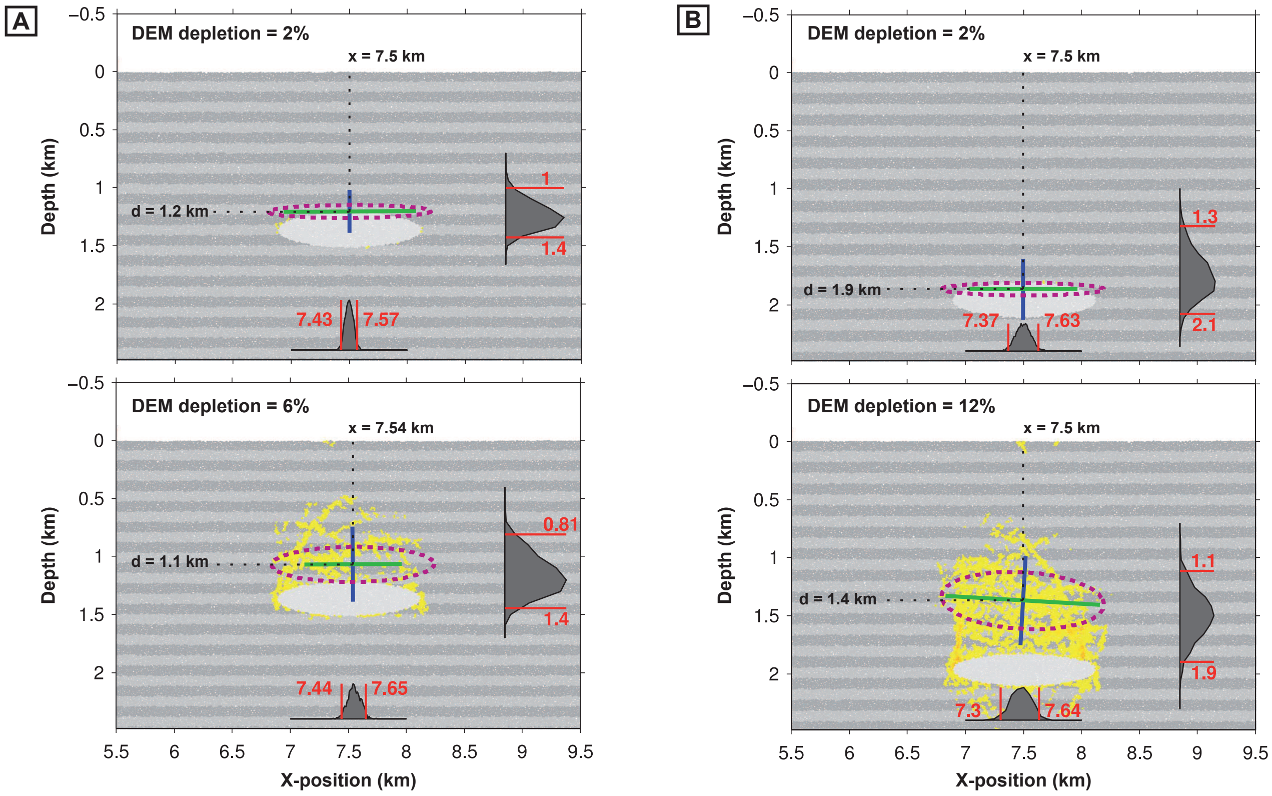

Figure S9: Results for decreased depth/diameter ratio of the DEM magma body. Shown here are optimum two-plane sources for cumulative displacements induced by depletion of the DEM magma body at a depth of **(A)** 1200 m (depth/diameter = 1) and **(B)** 1800 m (depth/diameter = 1.5). Top row: elastic strain (2% depletion); Bottom row: non-elastic strain immediately prior to failure through to the surface (6 and 12% depletion). Likelihood distributions for the depth and lateral position of the source's centre are shown in the dark-grey histograms. Red bars on the histograms show the 2.5 and 97.5 percentiles.
